# A new registration algorithm of electronic portal imaging devices images based on the automatic detection of bone edges during radiotherapy

**DOI:** 10.1038/s41598-020-67331-9

**Published:** 2020-06-24

**Authors:** Cheng Chen, Chaoyan Wu, Yahua Zhong, Conghua Xie, Yunfeng Zhou, Hui Liu, Jun Zhang, Jiuling Sheng, Dazheng Jiang, Hongli Zhao, Haijun Yu

**Affiliations:** 1 Department of Radiation and Medical Oncology, Hubei Province Cancer Clinical Study Center, Hubei Key Laboratory of Tumor Biological Behaviors, Zhongnan Hospital of Wuhan University, Wuhan, 430071 China; 2 Department of Traditional Chinese Medicine, Zhongnan Hospital of Wuhan University, Wuhan, 430071 China

**Keywords:** Radiotherapy, Applied mathematics

## Abstract

The precision and efficiency of the registration of megavolt-level electronic portal imaging devices (EPID) images with the naked eye in the orthogonal window are reduced. This study aims to develop a new registration algorithm with enhanced accuracy and efficiency. Ten setup errors with different translation and rotation were simulated with the phantom. For each error, one set of simulated computer tomography images and EPID images were acquired and registered with the traditional and the new method. The traditional method was performed by two senior physicists with the Varian Offline Review software. The new method is basing on the comparison of the precise contours of the same bone structure in the digital reconstruction radiography images and the EPID images, and the contours were fitted with an automatic edge detection algorithm based on gradient images. The average error of the new method was decreased by 44.44%, 28.33%, 49.09% in the translation of X, Y, and Z axes (The traditional vs. the new: X axes, 0.45 mm vs. 0.25 mm; Y axes, 0.75 mm vs. 0.35 mm; Z axes, 0.55 mm vs. 0.28 mm), 42.86% and 40.48% in the rotation of X and Z axes (The traditional vs. the new: X axes, 0.49° vs. 0.28°; Z axes, 0.42° vs. 0.25°), respectively. The average elapsed time in the new method was reduced by 11.14% (The traditional vs. the new: 44 s vs. 39.1 s). The new registration method has significant advantages of accuracy and efficiency compared with the traditional method.

## Introduction

Image-guided radiation therapy (IGRT) has become an important method of radiotherapy technology. The anticipated benefit of IGRT is realized basing on online confirmation of the registration at the time of radiotherapy. Megavoltage (MV) electronic portal imaging device (EPID) has been still widely used as an online verification tool for the treatment field and dosimetry in radiation therapy, although the emergence of kilovoltage (kV) imager mounted on a linear accelerator^1–7^.

Although cone-beam computed tomography (CBCT) affords much valuable soft-tissue information when the patient is on the treatment board during radiotherapy, there are some primary concerns about with this imaging method: the additional imaging dose and time, an X-ray source that is offset 90° from the treatment beam and the accuracy of localization using various registration algorithms. The imaging dose of EPID can be easily merged into the treatment dose, which is impossible to the CBCT. Several researches^8,9^ have been carried to investigate the excess dose induced by imaging received by patients during IGRT using KV-CBCT, which is associated with the risk of the second cancer^10^, especially for children and should be minimized. Hammoud et al.^11^ have reported extra doses to the skin from 1.5 to 2.5 Gy and the body from 1.3 to 1.8 Gy for 42 fractions of prostate treatments detected by thermoluminescent dosimeter (TLD). Ding's research^12^ showed a full rotation pelvis scan could induce an extra dose of about 1 to 2 cGy. The high price of the CBCT-based IGRT system also limits the widespread use in the clinic.

The EPID-based IGRT system is cheaper than the CBCT system. The use of the therapeutic beam during the imaging of EPID can avoid the problem of inconsistency between the beam center and the imaging center and enable anatomical visualization from the beam's-eye-view^13^. So there is still a broad application in clinical practice. However, there is an obvious limitation for EPID-based IGRT. The radiotherapists must determine the accuracy of the registration results from the automatic registration or manual registration of EPID images. If the registration error is significant, re-registration or manual adjustment is needed until the registration result meets the treatment requirements. Therefore, the accuracy of the registration judgment is an essential factor in determining the precision of EPID-based IGRT. The image contrast suing the MV treatment beam is formed predominantly by Compton interactions, hence showing electron density variations, while contrast in kV X-ray beams is mostly generated by photoelectric interactions. Thus, the images from EPID are intrinsic poor in the fields of contrast and information because the beam from linear accelerators is shaped for treating cancer rather than for imaging. Due to the low contrast of the megavolt image of EPID, the bony structure in the image is not easily distinguishable from the soft tissues, such as muscle and fat. Besides, the quality of digital reconstruction radiograph images generated from a CT-electron density conversion curve by the planning system is not enough for accurate registration. The enhancement of the contrast between the soft tissue and the bone by defining a special CT value-to-density conversion curve is still time-consuming and inefficient. Thus the factors above significantly affect the accuracy of the manual judgment of the EPID registration results. A new method for judging the registration results of megavoltage EPID images is greatly needed.

At present, relevant researches mainly focus on improving EPID image quality with image enhancement technology. Some efforts have been committed to the improvement of EPID image quality through the amendments of detector design. Yip et al.^2^ attempted to improve the quality of EPID images with a combination of four conventional EPID layers, which made a significant enhancement of tracking accuracy of poorly visible objects. Masatsugu Hariu et al.^14^ improved the EPID image quality by reducing the Compton scattered photons estimated at each point through the Monte Carlo simulation. Joerg Rottmann^13^ uses a design with four detection layers, which improves image quality compared to traditional single-layer EPID. Basing on the enhancement of the image by histogram equalization, Chen Yuan-Po^15^ enhanced the partial image quality with the help of the retinex algorithm and improved the contrast of the image. HR Tizhoosh^16^ combined fuzzy techniques with neural network algorithms and used prior knowledge to enhance megavoltage images. However, global enhancement methods were taken in some studies above. Although enhancing the specific bone edges, the textures around the bone edges are also enhanced, which reduces the precise judgment of the bone edges. Some results were failed, and some proposed methods are not suitable for online image alignment.

To develop a precise and effective method of the judgment of the registration results, here we proposed a new way for judging the registration results of EPID images based on edge extraction without permanently altering either the photon source or the detector. DRR and EPID images of ten intentional setups of the translation and rotation simulated with a head and neck phantom were acquired, the bone edges of the same bone in the two images were calculated with an automatic edge detection algorithm based on gradient images. The new registration methods can be performed by comparison the bone edges of the same bone in EPID and DRR images. The results in the present study indicated that the new registration is an effective and time-saving method compared the traditional method.

## Results

### Polynomial curve fitting of the marker point

**Figure 1:**
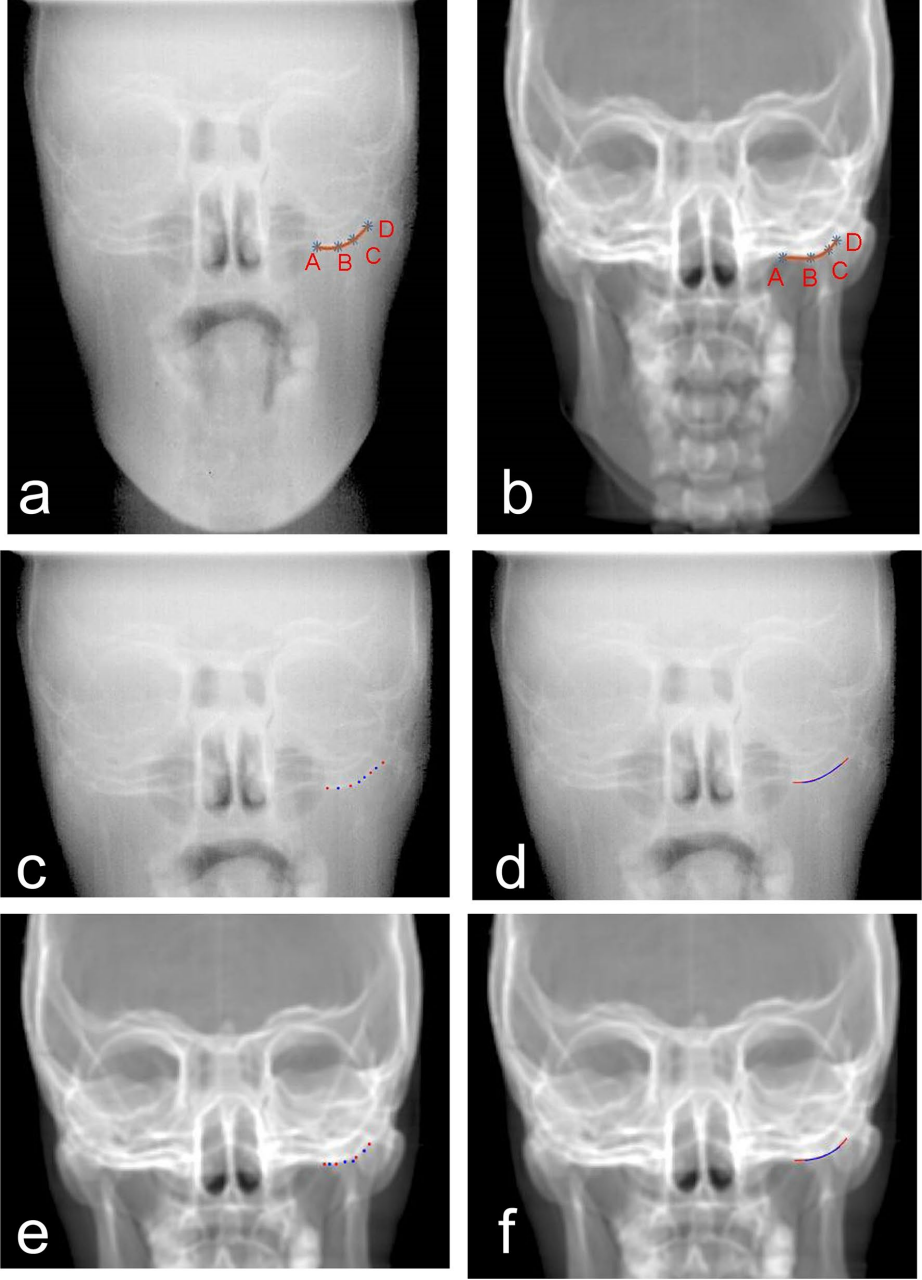
Cubic polynomial curve fitting basing on four markers. Cubic polynomial curve fitting from EPID (**a**) and DRR (**b**) images of the same phantom in the same position. Repeated cubic polynomial curve fittings from the EPID images of the same phantom in the same position based on two different groups four markers in EPID and DRR images (Eight different points in (**c**) for the EPID image and (**e**) for DRR image, red and blue curves in (**d**) for the EPID image and (**e**) for DRR image).

The polynomial curve fitting curves of the four markers in the phantom with ten specified positions were successfully fitted in the DRR images and the EPID images. The result for one example is shown in Fig. 1a and b. Four red and four blue points were randomly selected in the same bone from the EPID (Fig. 1c) and DRR (Fig. 1e) images, respectively. Then the curves from two group points were piloted. The two fitting curves from the two groups of points were identical (Blue and red fitting curves showed in Fig. 1d for the EPID image and Fig. 1f for the DRR image). Thus, the cubic polynomial curve can fit the edge of the bone repeatedly, which makes the new registration available.

### The new registration method enhanced the accuracy of the registration

**Figure 2:**
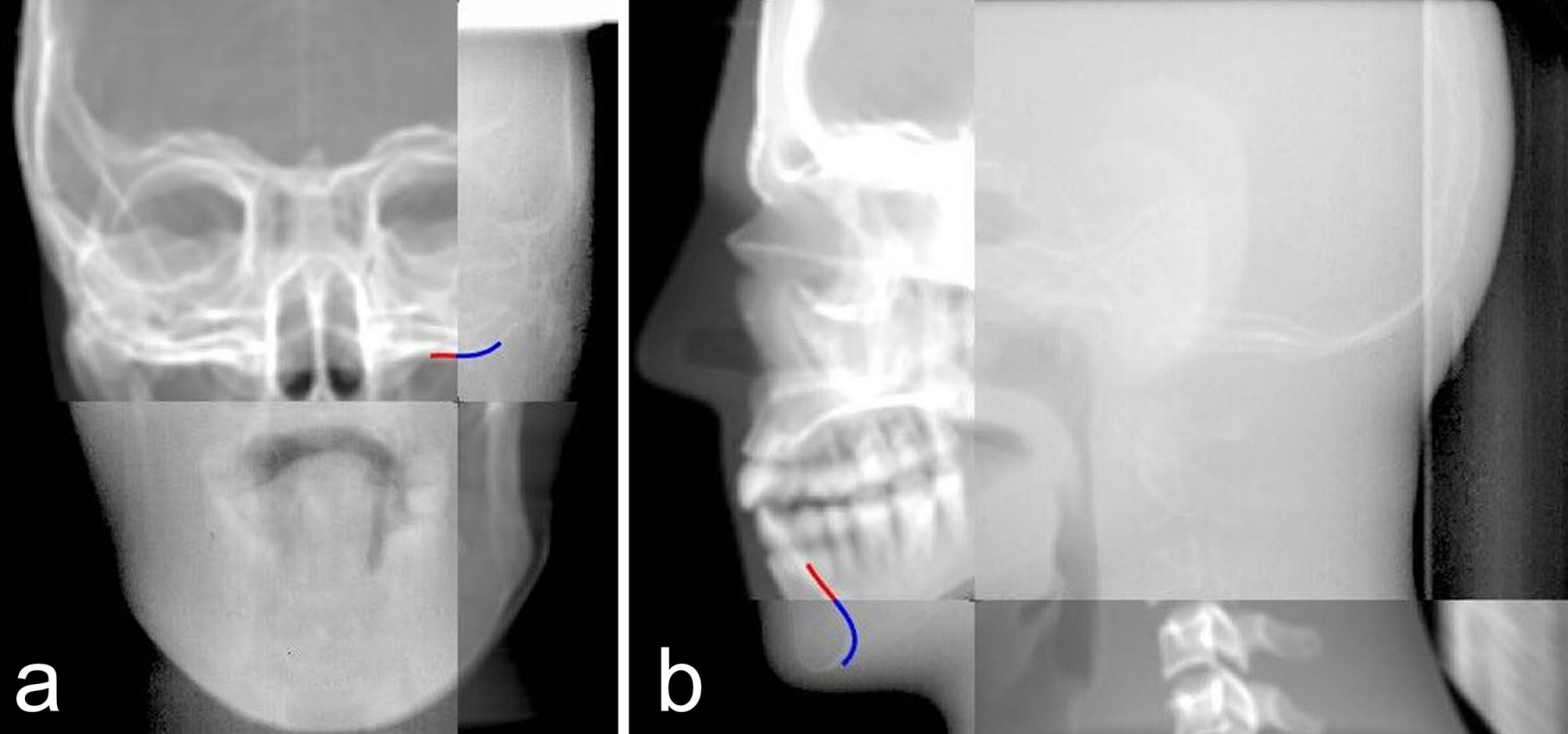
Figures of the registration result of the new registration. (**a**) Results of anteroposterior registration, (**b**) results of lateral registration.

**Figure 3:**
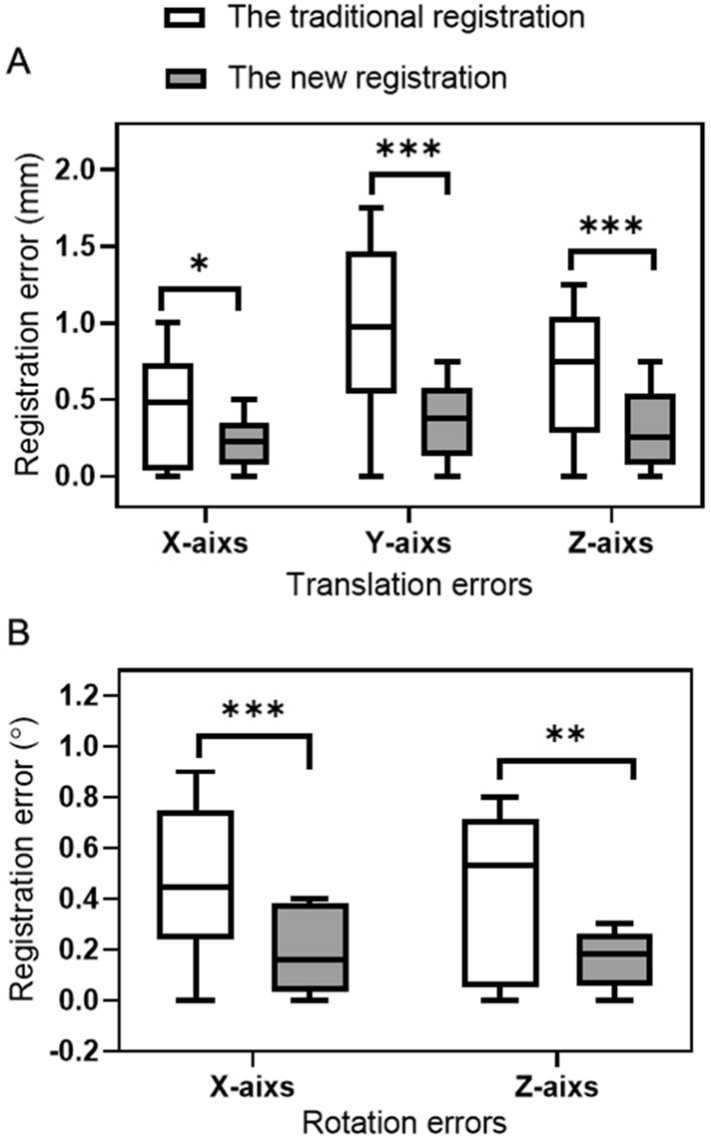
Quantitative comparison of the accuracy between the two methods of registration. The translation errors (**A**) and the rotation errors (**B**) are compared between the two registration methods. *P < 0.05, **P < 0.01, ***P < 0.001.

The new registration result judgment method was based on identifying the bony structure through two fitting curves with different colors. The coincidence of two curves represents the registration results on the split window (Fig. 2). The error of different registration results is shown in Table 1 and Fig. 3. There is a significant difference in the mean errors of the translation registration between the two registration methods in three dimensions (For X-axis: Traditional method vs. New method: 0.45 mm vs 0.25 mm, *P* < 0.05, for Y-axis: Traditional method vs. New method: 0.75 mm vs. 0.35 mm, *P* < 0.001, For Z-axis: Traditional method vs. New method: 0.75 mm vs 0.55 mm, *P* < 0.001). The differences of mean rotation registration errors in X and Z axis between the two methods are significant (In X-axis: Traditional method vs. New method: 0.49° vs. 0.28°, *P* < 0.001, in Z-axis: Traditional method vs. New method: 0.42° vs. 0.25°, *P* < 0.01). These data above indicate that the accuracy of the new registration method is better than the currently used registration method.

**Table 1:** Quantitative comparison of the accuracy between the two methods of registration**.**

Parameters	Error_min_		Error_max_		Error_mean_		*t* value	*P* value
	1	2	1	2	1	2		
**Translation (mm)**								
X-axis	0	0	1.00	0.5	0.45	0.25	4.000	0.003
Y-axis	0	0	1.75	0.75	0.75	0.35	2.848	0.019
Z-axis	0	0	1.25	0.75	0.55	0.28	3.973	0.003
**Rotation (°)**								
X-axis	0	0	0.9	0.4	0.49	0.28	2.848	0.024
Z-axis	0	0	0.8	0.3	0.42	0.25	2.321	0.047

*Note* Error_min_, Error_max,_ and Error_mea_ are the minimum, maximum and average values of registration errors of the two judgment methods, respectively. Method 1 and 2 represent the traditional registration method and the new registration method, respectively.

### The new registration method shortened the elapsed-time of registration

The elapsed time of different registration methods was measured in the present study. Table 2 showed the results of the statistical analysis of the elapsed-time of two registrations. The average elapsed-time of the traditional and new registration methods is 44 s and 39.1 s, respectively (Traditional method vs. New method: 39.1 vs. 44 s, *P* = 0.051). The new registration method showed the tendency of more shortened elapsed-time but without the statistical significance.

**Table 2:** Comparison of the elapsed-time of the two registration methods.

Parameters	Methods 1 (s)	Methods 2 (s)	t value	P value
Time_min_	25	32		
Time_max_	63	49		
Time_mean_	44	39.1	2.255	0.051

*Note* Method 1 and method 2 represent the traditional registration method and the new registration method.

## Discussion

Electron field imaging devices are currently used in many clinical applications. Compared with cone-beam computed tomography (CBCT) KV imaging, the image quality of EPID is poor, especially the unsharp edges of bony structures. The deficiency of the precise discriminability of bony borders for EPID has brought great difficulties to the current artificial judgment of the registration result through the naked eye, which reduces the accuracy of image-guided radiotherapy.

To settle this problem, we explored a method for extracting the edge of the bone structure of EPID megavoltage images. The original blurred edges were extracted and delineated, which well benefited the judgment of the registration results. This new method significantly reduced the judging errors from different technicians with the enhancement of their efficiency. According to our experimental results, the average error of the new registration method in the X, Y, and Z axes was reduced by 44.4%, 53.3%, and 49.1%, respectively, with the shorten elapsed-time.

It will take some time for technicians to roughly outline the edge points of bony structures in the EPID and DRR images and find the accurate edges by optimizing the algorithm in the new registration method before judging the degree of coincidence of the bony structure edges. However, it is effortless and less time-consuming for judging the degree of coincidence between the two curves if the corresponding bone edges in the EPID and DRR images have been determined. The searching and matching range within 3 mm in our new optimization algorithm will significantly reduce the region of search and thus reduce the optimization time. The traditional widely used judging method of the registration results has the opposite rationale. Although it can directly judge the degree of coincidence of the bony structure at the beginning, the ambiguous edges are hard to distinguish for technicians, which finally increases the judgment time. Therefore, the average time-consuming of the new registration method is less than that of the currently used registration results.

Several studies showed that different algorithms were taken for the registration between DRR images and EPID images or port films. The DRrPortRegistration was used for the registration between DRR images and port films, the mean absolute average errors in the DRrPortRegistration are equal to 0.2 degrees, 0.75 mm in the X-direction, and 0.81 in the Y-direction^17^. Cheong^18^ reported that the CLAHE algorithm is the best effective step for the quality enhancement on the MV EPID image in terms to tumor tracking after comparing the individual contribution of deblurring, contrast improvement (CLAHE) and denoising, the detection errors in the contrast improvement group are 2.9 mm in the Y direction and 2.7 mm in the X-direction. The DRrPortRegistration and CLAHE algorithms showed an inferior accuracy than the present registration. The rationale of the present study is based on determining the border of the bone with the maximal gradient value of bone border. The traditional methods of deblurring, contrast improvement and denoising generally result in the whole change of the bone and soft tissue, which frequently dims the precise border of the bone.

One of the limitations of this study is that the data and the results were not confirmed in the patient daily treatment processes. The differences between phantoms and patients may impact some uncertain effects on the present conclusion. The second limitation is that the Y-axis rotation errors are not taken into account in the present study. In the future, further study about the new registration result judgment method will be tested in the real practice of patients.

## Conclusion

In summary, the new registration result judgment method proposed here for EPID images is feasible. The new registration result judgment method is more accurate and less time-consuming than the currently used methods, which may facilitate the clinical practice of the new method in the future.

## Materials and methods

### EPID and linear accelerator (LINAC) features

**Figure 4:**
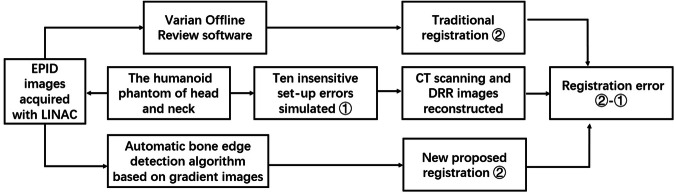
Schematic workflow for the evaluation of the two registration methods between DRR and EPID images during IMRT.

A diagram of the workflow of the present study is indicated in Fig. 4. In this study, experimental measurements were made using Varian Unique linear accelerator (Varian Medical Systems, Inc.) in Zhongnan Hospital of Wuhan University. The aS1000 EPID (Varian Medical Systems) is mounted on the LINAC gantry with 140 cm for the source-to-detector distance. The physical size of the EPID was as follows: the EPID had a sensitive area of 40 cm × 30 cm in size, and the effective pixel size was 0.039 cm × 0.039 cm. Each exposure used in this study was acquired using at least 2 MU at a nominal dose rate of 300 MU min^−1^. The images were stored in the Heimann information system (HIS) format for analysis using in-house code written in the interactive display language (ITT Visual Information Solutions).

### Intentional setup errors simulation with a phantom

A phantom experiment was introduced to quantify the accuracy of the EPID setup. The humanoid phantom (CIRS 711-HN, CIRS Tissue Simulation Technology) was comprised of a human skull, made of high phosphorus, high calcium resin, with lower density materials filling the cranium and the oral and nasal cavities. Ten intentional setup errors (10 translations and rotations) were simulated by introducing off setup to ± 7 mm in three principal axes (X: medio-lateral, Y: anterior–posterior, Z: cranio-caudal )and 0° to ± 3° in yaw. The detailed setting errors were shown in Table 3. The phantom was placed on an in-house developed "breathing" board with a hinge on the LR axis at the level of the abdomen and an amplitude of 1 cm at the level of the clavicles. The phantom was set up isocentrically and imaged using 25 × 25 cm^2^ anterior fields. CT scans of the phantom in ten specific setups were acquired with a large aperture simulation CT scanner (Siemens Somatom Sensation). DRR images were created with an Eclipse (V11.0, Varian Medical Systems) treatment planning system. EPID images of the phantom at the 0° and 90° of the gantry were gained with Varian Unique linear accelerator in ten specific setups.

**Table 3:** Displacement applied in translation and rotation for measurement of residual error from the IGRT system.

Simulated error type	Translation (mm)			Rotation (°)	
	X-axis (medio-lateral)	Y-axis (anterio-posterio)	Z-axis (cranio-caudal)	X-axis	Z-axis
1	3	− 3	2	− 1	− 2
2	− 1	5	− 2	− 1	− 2
3	2	1	6	2	1
4	2	− 3	− 2	3	− 2
5	5	2	− 1	− 1	0
6	1	6	− 2	1	− 2
7	− 2	1	3	− 2	− 1
8	3	5	2	− 2	− 3
9	4	0	− 3	− 2	0
10	− 7	− 2	0	1	3

### Traditional EPID Image registration method

**Figure 5:**
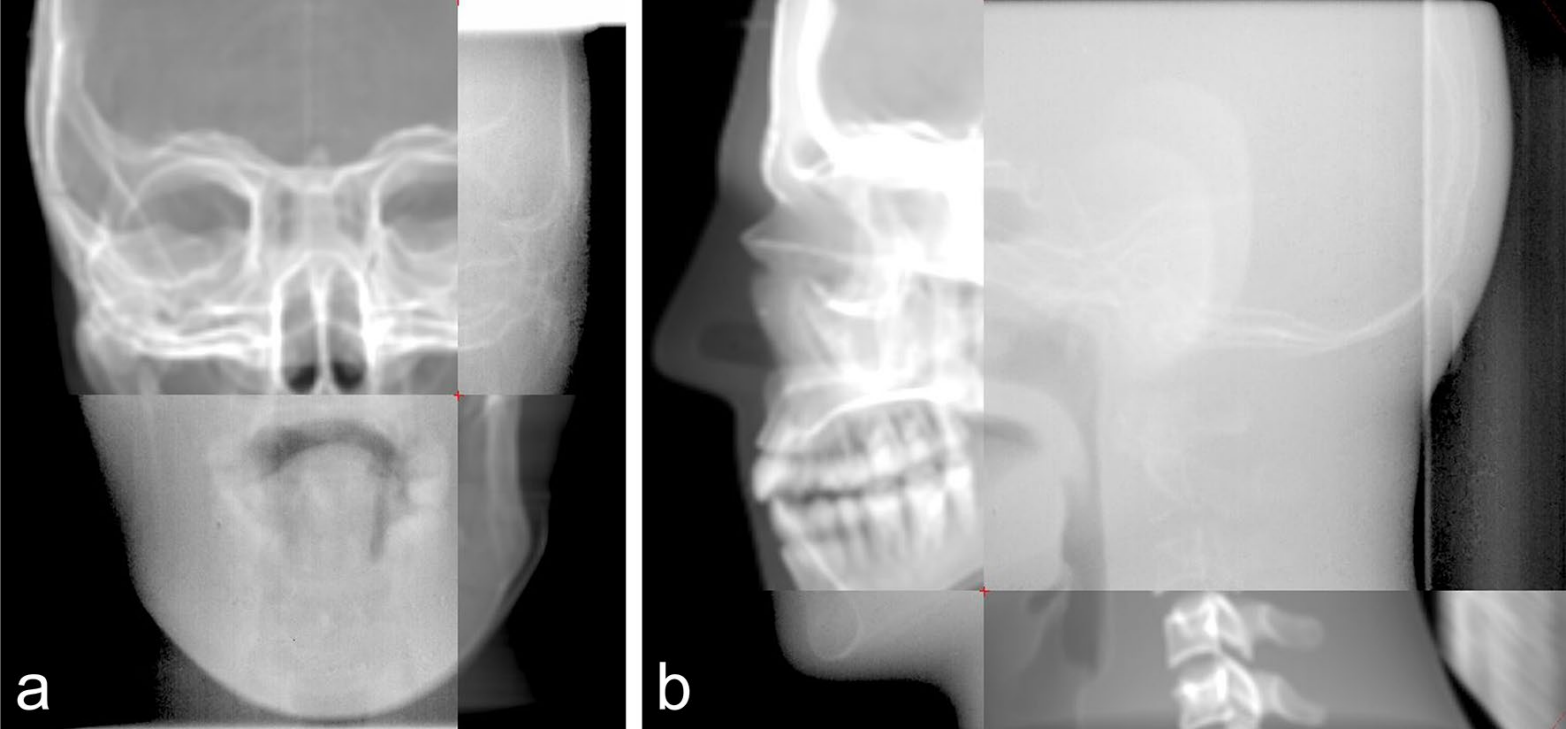
The traditional registration method of EPID and DRR images through the split window: (**a**) fusion images of anteroposterior view, (**b**) fusion images of lateral view.

The traditional method of registration between EPID images and DRR was briefly introduced as follows. For each phantom with ten specified positions, an orthogonal pair of images was generated from DRR and the electronic portal images (EPIs). Manual matching of the corresponding EPIs and DRRs using horizontal and vertical image translations to align corresponding markers as visualized in each of them optimally was performed offline by the two trained senior radiotherapy physicists using Varian Offline Review software (Varian Medical Systems). The obtained image translations (in pixels) were automatically converted to left–right, cranio-caudal and anterior–posterior couch shifts (in mm) based on the orientation and pixel size of the EPIs. The detailed method was shown in Fig. 5.

### New proposed EPID registration method

**Figure 6:**
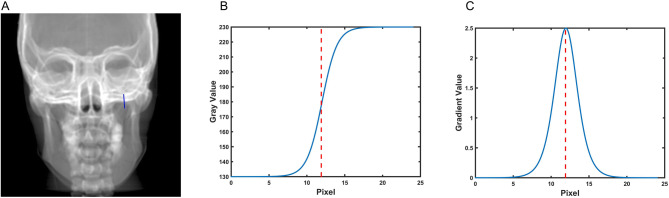
Example of determining of one-point border of a bone. (**A**) A random point near the border of the bone and the vertical line passing through the point. (**B**) Gray value of each pixel in the vertical line. (**C**) Gradient value of each pixel in the vertical line. The pixel of maximal Gradient value is identified as the corresponding point of the bone border.
In the EPID and DRR images, the approximate positions of the edges are identified manually by points A, B, C, and D along the edges of the same selected bony structure (showed in Fig. 1).
Perform a cubic polynomial curve fitting of the four markers. The cubic polynomial curve fitting function is as follows:
  2 $$ {\text{f}}\left( {\text{x}} \right) = {\text{ax}}^{{\text{3}}}  + {\text{bx}}^{{\text{2}}}  + {\text{cx}} + {\text{d}} $$
Calculate the sum of gradients from point A to point D on the fitting curve through the gradient image formula.
The sum of gradients is taken as the objective function, the x and y coordinates of points A, B, C, and D are optimized parameters. To find the maximum value of the objective function, each optimized parameter is searched according to the step size of a single-pixel within the range of [− 3, + 3] mm around the initial point. Taking into account the advantages of the capability of the global optimization search and the convergence speed of the local optimization, we introduced Particle Swarm Optimization (PSO) and Powell optimization algorithm. The hybrid optimization algorithm is to find the maximum value of the objective function, obtain the accurate osseous structure edge, and highlight the EPID and DRR images with different colored curves (showed in Fig. 2).
(6) The accuracy of the registration result is determined by the coincidence degree of the two different color curves on the registration fused images.

The new approach of the EPID registration is basing on the definition of the bone border. We proposed an automatic edge detection algorithm based on gradient images to more accurately identify the boundary of the bony structure. The specific procedures are as follows:

Calculate the gradient image of EPID and DRR images. The gradient formula is as follows:
  1 $$ {\text{G}}\left( {{\text{x}},{\text{ y}}} \right) = dx\left( {i,j} \right) + dy(i,j) $$
  In the formula, *dx(i, j)* = I(i + 1,j) − I(i, j), dy(i, j) = I(i,j + 1) − I(i, j), I Is the grayscale value of the pixel, *(i, j)* is the pixel's coordinates
  The detailed procedures are shown in Fig. 6. Briefly, a point along the border of the bone was localized, and a vertical line passing through the point was drawn (Fig. 6A). Then the gray value (Fig. 6A) and gradient value (Fig. 6C) of each pixel of the vertical line are calculated. The pixel of the maximal gradient value is identified as the point of the border of the bone.

### The verification of the registration results

The head phantom was applied to simulate the real patient's setup error. First, the isocenter of the phantom was set up with the Varian Acuity simulation (Varian Medical Systems). The phantom was scanned on Siemens Somatom Sensation open (Siemens) with a 2 mm thickness per layer, and the images were transferred to the treatment planning system (Varian Eclipse 13.5 planning system, Varian Medical Systems, Inc.) to generate the DRR. Then the phantom was fixed on the Varian Unique Accelerator. Ten cases of setting error in the dimensions of the X-axis (left and right), Y-axis (head), and Z-axis (up and down) were simulated by moving the treatment couch. The phantom anteroposterior and lateral images of each setting error were obtained with the EPID of the Varian Unique Accelerator.

The mutual information method was used to register the DRR reference image and the EPID images. The mutual information formula is as follows

3 $$  {\text{I}}\left( {{\text{A}},{\text{ B}}} \right) = H\left( A \right) + H\left( B \right) - H(A,B)  $$

In the formula, *H (A)* and *H (B)* are the entropies of the images A and B, respectively, and the units are bits; *H (A, B)* is the joint entropy^6^ of the images A and B.

The traditional and new methods were used to judge the registration results, and the position of the image is adjusted until the registration results were considered to meet the clinical requirements by medical physicists and radiotherapy oncologists. The elapsed time and the final setup error value were recorded. The difference between the setup error values obtained by the methods and the setting error values was calculated.

### Software implementation and statistical analysis

In the present study, Matlab R2014b software was used to define the bony border structure. Graphpad Prizm 5.0 software was introduced to perform a paired t-test on the results of 10 simulated setup error experiments. Significance was defined as *P* < 0.05.

## Data Availability

Data produced and processed in this study are included in the published article. The more datasets can be acquired from the corresponding author upon appropriate purposes.
